# Social, Economic, and Regional Determinants of Mortality in Hospitalized Patients With COVID-19 in Brazil

**DOI:** 10.3389/fpubh.2022.856137

**Published:** 2022-03-31

**Authors:** Waldecy Rodrigues, Humberto da Costa Frizzera, Daniela Mascarenhas de Queiroz Trevisan, David Prata, Geovane Rossone Reis, Raulison Alves Resende

**Affiliations:** ^1^Program of Computational Modelling, Federal University of Tocantins, Palmas, Brazil; ^2^Program of Institute of Regional Development, Federal University of Tocantins, Palmas, Brazil; ^3^Insper—Institute of Education and Research, São Paulo, Brazil

**Keywords:** social factors, economic factors, regional factors, mortality in hospitalized, COVID-19

## Abstract

On May 10, 2021, Brazil ranked second in the world in COVID-19 deaths. Understanding risk factors, or social and ethnic inequality in health care according to a given city population and political or economic weakness is of paramount importance. Brazil had a seriousness COVID-19 outbreak in light of social and economic factors and its complex racial demographics. The objective of this study was to verify the odds of mortality of hospitalized patients during COVID-19 infection based on their economic, social, and epidemiological characteristics. We found that odds of death are greater among patients with comorbidities, neurological (1.99) and renal diseases (1.97), and immunodeficiency disorders (1.69). While the relative income (2.45) indicates that social factors have greater influence on mortality than the comorbidities studied. Patients living in the Northern macro-region of Brazil face greater chance of mortality compared to those in Central-South Brazil. We conclude that, during the studied period, the chances of mortality for COVID-19 in Brazil were more strongly influenced by socioeconomic poverty conditions than by natural comorbidities (neurological, renal, and immunodeficiency disorders), which were also very relevant. Regional factors are relevant in mortality rates given more individuals being vulnerable to poverty conditions.

## Introduction

On December 31, 2019, the first cases of the international rapidly expanding SARS-CoV-2 virus were reported to the World Health Organization (WHO), which until then was defined as a “pneumonia of unknown origin.” After migrating beyond China's borders, the WHO established that it was a pathological outbreak caused by the Severe Acute Respiratory Syndrome Coronavirus 2 (SARS-CoV-2) virus, which caused Severe Acute Respiratory Syndrome (1, 2). Months later, on 11 March, 2020, the WHO declared a pandemic, since the virus had spread exponentially to countries all over the world (3, 4). Brazil became the third most affected country in the world, in terms of cases, and second in deaths, from COVID-19 (5).

During the SARS-CoV-2 (COVID-19) pandemic, social and economic factors shown evidence for national and global social disparities (6–8). Lower-income populations suffered most from the systems employed for combating the pandemic, e.g., difficulties in maintaining social lockdowns due to employment and income factors, and less access to health care and basic sanitation (9).

Brazil is an extremely important country for carrying out impact study on the COVID-19 pandemic, since it had a serious outbreak, given its social and economic circumstances, and its complex racial composition. The objective of this study was to statistically verify the mortality rates of patients hospitalized during COVID-19 infection, based on their economic, social, and epidemiological characteristics, and by comparing the main findings with other international studies on the theme (10, 11).

Baqui et al. (11) presented a cross-sectional study with data from the same database source used in this study, when Brazil had 3,254 deaths. It gave results on the mortality factors for COVID-19 including symptoms, age, sex, ethnicity, and comorbidities. Subsequently, THE LANCET (12) suggested that research on mortality from COVID-19 should consider social and biological factors, but also pay attention to ethnic and socioeconomic disparities among patients, emphasizing that the lack of associations between ethnicity and mortality from COVID-19 is of great concern, highlighting a research gap. To contribute to this concern, the main issue in this research is to answer the question: in addition the comorbidities, were social factors important determinants of the COVID-19 mortality in Brazil?

This study analyzed variables like comorbidities, age, gender, ethnicity, and geographic region of residence, separately, similar to the cited works. Data were collected from the Hospitalized Severe Acute Respiratory Syndrome (SRAG) database, managed by the Ministry of Health (13), when Brazil had 331,435 COVID-19 deaths, from February 22, 2020 to May 10, 2021.

Additionally, this study carefully analyzed social factors. We used indices for level of education, gender, age, and race, considering the natural divisions made up of Brazilian states, provided by the IBGE Automatic Recovery System (SIDRA) from data from the National Household Sampling Survey (PNAD) (14). We were able to obtain evidence related to social factors and COVID-19. The importance of the subject is that it may reveal disparities among a patient's social condition, and the probability of the disease progressing to death.

## Methods

### Data Collection

Brazilian Ministry of Health maintains a system called Influenza Epidemiological Surveillance Information System (SIVEP-Gripe) (13), that contains a public dataset about Hospitalized Severe Acute Respiratory Syndrome (SRAG), from both public and private hospitals. In this study, we collect data from February 22, 2020, when the first case of hospitalization for COVID-19 was registered in Brazil, to May 10, 2021, when Brazil had a total of 1,956,350 epidemiological patient records for people who sought hospital care at all Brazilian states. At that time, Brazil had 331,435 COVID-19 deaths.

From that dataset were collected some variables, that are: age, gender, ethnicity, education level, comorbidities, case evolution (death or survival), the city, and the state where the patient lives. SRAG database also contains patient symptoms, in addition to the aforementioned information.

To compose our dataset, we collected indicators on social factor from the National Survey by Sample and Households-PNAD (2020) (14), that were characterized by age, race, gender, and education per state. These indicators were assigned to each patient according to their demographic characteristics and place of residence registered on SIVEP-Gripe. The dataset created allowed us to consider not only health factors to the analyze, but also social.

### Study Design

After the data collection, some variables were processed for this study. We created a new variable to set up the region where the patient lives and classified the ethnicity, according to the registered data.

Regarding the Brazilian states and regions, although Brazil is divided into five regions, the country was divided into two macro-regions in this study, using their characteristics, similar to the approach by Baqui et al. (11). The Northern macro-region encompasses the Northern and Northeastern regions, and the Central-South macro-region covers the Central-Western, Southeastern, and Southern regions.

This regional division was done for modeling and data analysis purposes for the chosen statistical models. There is a proportionality between the number of COVID-19 cases, the number of hospitalized and deaths with the population of the territorial macro-region, North and Central-South, so they can be compared using descriptive statistics. Also, this division was considered quite natural by Baqui et al. (11) since the socioeconomic factors are similar.

To analyze the odds of mortality among races, patients were grouped into two classes, as white or non-white. For the descriptive statistics, we used the names for races as defined by the IBGE defines as: White, Black, Asian, Indigenous, or Mixed-race.

**Figure 1:**
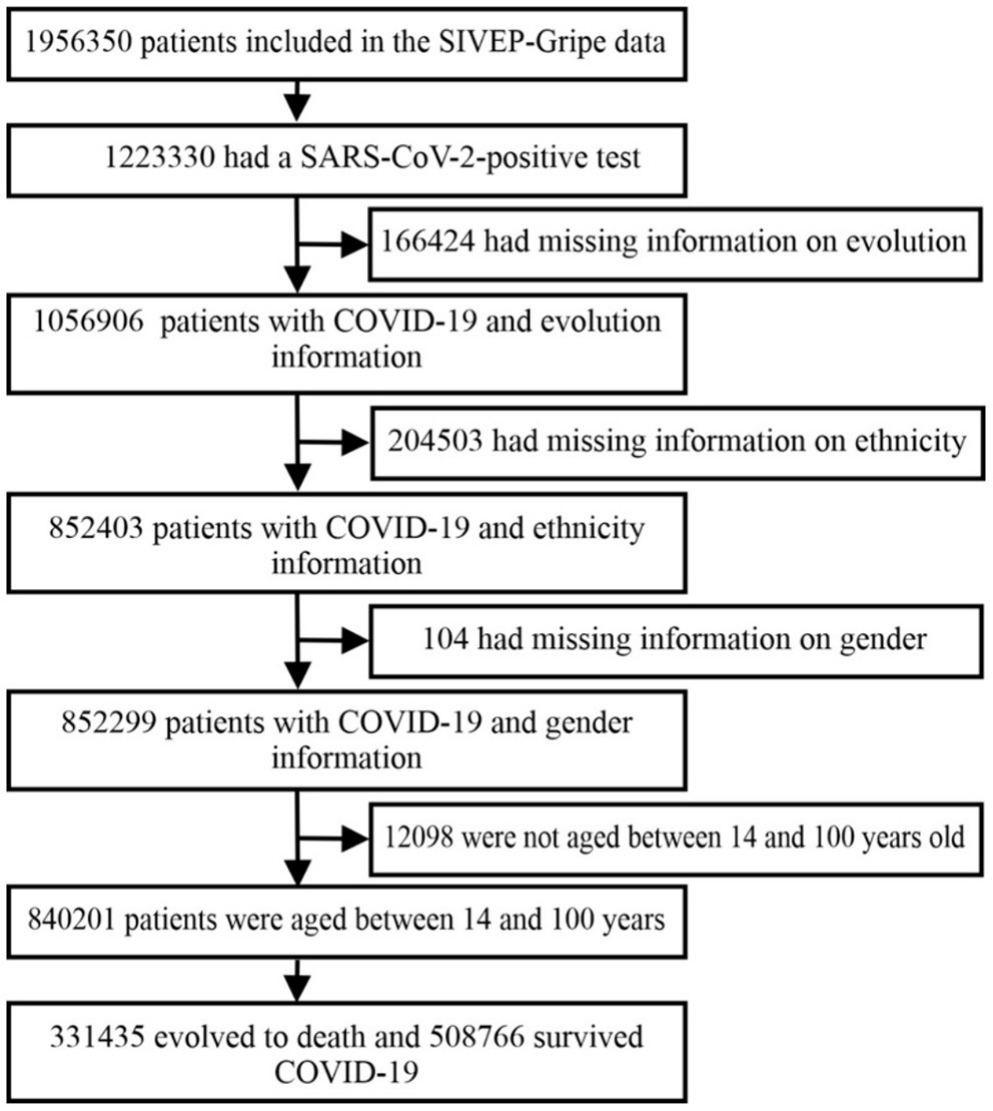
Flowchart of the SIVEP-Gripe database used in this analysis. SARS-CoV-2, severe acute respiratory syndrome coronavirus 2; SIVEP-Gripe, Influenza Epidemiological Surveillance Information System.

Thereafter, there were some selections in the databases to avoid null records following some criteria. Also, the social database (14) do not have data about people under 14 years old. Figure 1 shows data clippings from the initial database and the dataset used in this study. Of the total number of patients selected, 39.4% had died.

Of the 1,956,350 records collected from the SIVEP-Gripe SRAG, 1,223,330 (62%) patients were classified with COVID-19. 1,056,906 (54%) records had variable evolutions filled in. 852,403 (43%) records had correctly registered ethnicity. 852,299 (43%) listed genders. 840,201 (43%) patients were aged between 14 and 100 years. From the resulting database, 840,201 patients, 640,916 (76%) were from the Central-South macro-region, and 199,285 (24%) were from the Northern macro-region.

### Statistical Analysis

We used statistical data to quantify the impact of the COVID-19 pandemic on the number of deaths in Brazil. Our main question is to demonstrate the differences between regions, ethnicities, ages and, comorbidities of patients recorded in the database. The model shown the mortality rates based on variables with a fixed effect for the patients, which were comorbidities and social factors.

For this analysis, we used the logistic regression since the central question of this research is related to a dichotomous variable, whether the hospitalized individual evolved to death or not. The prediction analysis was based on the study by Rodrigues and Parreira (15).

Logistic regression is a useful tool for answering the odds of an event occurring. It can predict the binomial outcome of a dependent variable (target) using one or a set of independent variables (predictive) (16). Binary categorical variables are often used in empirical research in health sciences, such as History of Abortion: yes or no (17), Food Security Status: Insecure Food or Secure Food and Food Stability: <3 times or 3 times (18), Post-Traumatic Stress Disorder: yes or no (19).

The logit model is usually used when the dependent variable is binary, fundamentally to measure the probability of a given phenomenon. As the response of interest to the dependent variable, the binary logistic model can be represented as shown in Formula 1:


(1) $$\begin{array}{r} {\text{P}}_{\text{i}}=\text{E}\left(\text{Y}=1|{\text{X}}_{1\text{i}},,{\text{X}}_{\text{ni}}\right)={\text{e}}^{\text{b}0+\text{b}1\text{X}1\text{i}+\ldots .+\text{bnXni}}/\\ \;\left[1+{\text{e}}^{\text{b}0+\text{b}1\text{X}1\text{i}+\ldots .+\text{bnXni}}\right] \end{array}$$

The maximum likelihood was used to estimate the probabilities of deaths of individuals hospitalized with COVID-19. The objective was to maximize the likelihood function (or its logarithm), that is, to obtain (through an iterative process) the values of the model parameters in what way the probability of observing the values of Yi is the highest (maximum) possible.

In logistic regression, the sample size is fundamental (20). To apply the model, is also relevant to consider some aspects related to the statistical design. The method is sensitive to multicollinearity (high levels of correlation between independent variables), and the most common solution to overcome this limitation is to expand the sample (21).

The function is defined by logit (p) and is related to the probability of patient cases evolving to death, considering their comorbidities and social factors, i.e., using the relative income index. The p risk is calculated below:

The values are applied in the formula $logit\left(p\right)=\ln\left[\frac{p}{1-p}\right]$ and the odds of death is found as 1–*p*. Using this method, we calculated coefficients that indicate risks for COVID-19 case with Wald confidence intervals at 99% for the odds ratios.

## Results

**Figure 2:**
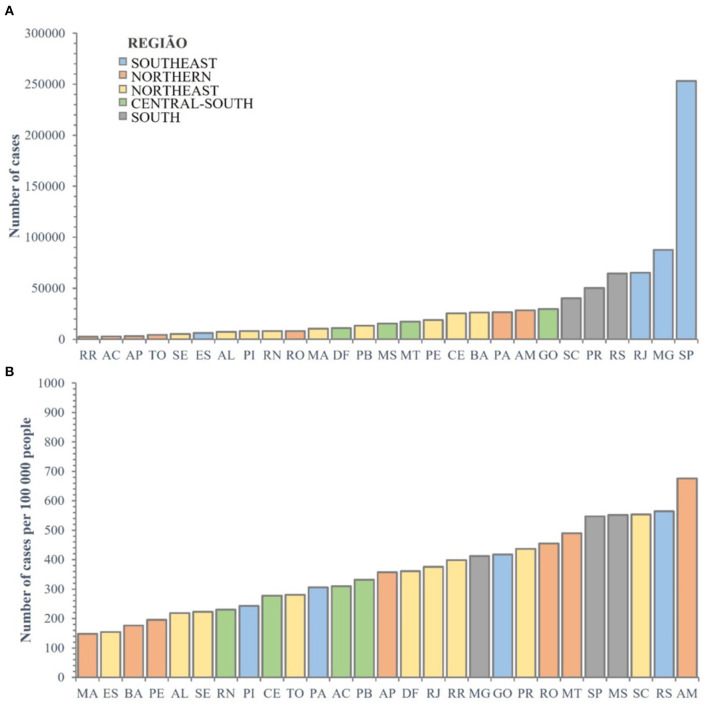
**(A,B)** Distribution of absolute number of cases among Brazilian states and the Federal District, and number of cases per 100,000 inhabitants. Cases = 840201. AC, Acre; AL, Alagoas; AM, Amazonas; AP, Amapá; BA, Bahia; CE, Ceará; DF, Distrito Federal; ES, Espírito Santo; GO, Goiás; MA, Maranhão; MG, Minas Gerais; MS, Mato Grosso do Sul; MT, Mato Grosso; PA, Pará; PB, Paraíba; PE, Pernambuco; PI, Piauí; PR, Paraná; RJ, Rio de Janeiro; RN, Rio Grande do Norte; RO, Rondônia; RR, Roraima; RS, Rio Grande do Sul; SC, Santa Catarina; SE, Sergipe; SP, São Paulo; TO, Tocantins.

The patients were registered in the system according to their city of residence. The absolute numbers are shown in Figure 2A, and the numbers of cases per 100,000 people are shown in Figure 2B.

Based on research from Baqui et al. (11), Tables 1, 2 show the demographic and comorbidity data between survivors and non-survivors, and their ethnic composition at each stage of the progression of COVID-19.

**Table 1:** Demographic characteristics and comorbidities among COVID-19 survivors and non-survivors.

	**Survivors**	**Non-survivors**
	**(*n* = 508,766)**	**(*n* = 331,435)**
North (*n* = 199,285)	106,266 (53.3%)	93,019 (46.7%)
**Age (years)**
14–50 (*n =* 58,747)	44,201 (75.2%)	14,546 (24.8%)
51–100 (*n =* 140,538)	62,064 (44.2%)	78,474 (55.8%)
**Gender**
Women (*n =* 88,225)	48,182 (54.6%)	40,043 (45.4%)
Men (*n =* 111,060)	58,084 (52.3%)	52,976 (47.7%)
**Ethnic group**
White (*n =* 28,998)	14,757 (50.9%)	14,241 (49.1%)
Mixed-race (*n =* 157,676)	848,85 (53.8%)	72,791 (46.2%)
Black (*n =* 8,481)	4,281 (50.5%)	4,200 (49.5%)
Asian (*n =* 2,821)	1,642 (58.2%)	1,179 (41.8%)
Indigenous (*n =* 1,309)	701 (53.6%)	608 (46.4%)
**Comorbidities**
Renal disease (*n =* 7,276)	2,405 (33.1%)	4,871 (66.9%)
Liver disease (*n =* 1,623)	586 (36.1%)	1,037 (63.9%)
Immunosuppression (*n =* 4,228)	1,822 (43.1%)	2,406 (56.9%)
Obesity (*n =* 11,017)	5,366 (48.7%)	5,651 (51.3%)
Neurological disease (*n =* 4890)	1,761 (36.0%)	3,129 (64.0%)
Pulmonary disease (*n =* 4,648)	1,663 (35.8%)	2,985 (64.2%)
Hematologic disease (*n =* 1,198)	530 (44.2%)	668 (55.8%)
Diabetes (*n =* 50,146)	22,397 (44.7%)	27,749 (55.3%)
Cardiovascular disease (*n =* 60,834)	27,351 (45.0%)	33,483 (55.0%)
Central-South (*n =* 640,916)	402,500 (62.8%)	238,416 (37.2%)
**Age (years)**
14–50 (*n =* 183,849)	152,460 (82.9%)	31,389 (17.1%)
51–100 (*n =* 457,067)	250,040 (55.1%)	207,027 (44.9%)
**Gender**
Women (*n =* 288,383)	183,063 (63.5%)	105,320 (36.5%)
Men (*n =* 352,533)	219,437 (62.2%)	133,096 (37.8%)
**Ethnic group**
White (*n =* 406,146)	256,977 (63.3%)	149,169 (36.7%)
Mixed-race (*n =* 188,455)	118,534 (62.9%)	69,921 (37.1%)
Black (*n =* 37,682)	21,438 (56.9%)	16,244 (43.1%)
Asian (*n =* 7591)	4,870 (64.2%)	2,721 (35.8%)
Indigenous (*n =* 1,042)	681 (65.4%)	361 (34.6%)
**Comorbidities**
Renal disease (*n =* 23,699)	9,464 (39.9%)	14,235 (60.1%)
Liver disease (*n =* 5,426)	2,379 (43.8%)	3,047 (56.2%)
Immunosuppression (*n =* 15,469)	7,352 (47.5%)	8,117 (52.5%)
Obesity (*n =* 53,845)	30,364 (56.4%)	23,481 (43.6%)
Neurological disease (*n =* 25,113)	9,984 (39.8%)	15,129 (60.2%)
Pulmonary disease (*n =* 25,134)	10,856 (43.2%)	14,278 (56.8%)
Hematologic disease (*n =* 4,540)	2,290 (50.4%)	2,250 (49.6%)
Diabetes (*n =* 158,467)	84,854 (53.5%)	73,613 (46.5%)
Cardiovascular disease (*n =* 225,237)	121,774 (54.1%)	103,463 (45.9%)

**Table 2:** Ethnic characteristics of patients at each stage of hospitalization from COVID-19.

	Brazilian population*	**Hospital admission**	**ICU admission**	**Death**	**Death/ hospitalization**	**Death (Not ICU)**	**Death (ICU)**
**Northern (*****n** **=*** **199,285)**							
White	27.8%	28,998 (14.6%)	10,173 (16.2%)	14,241 (15.3%)	49.1%	6,867 (14.3%)	7,374 (16.4%)
Mixed-race	61.5%	157,676 (79.1%)	48,667 (77.6%)	72,791 (78.3%)	46.2%	37,960 (79.0%)	34,831 (77.4%)
Black	8.8%	8,481 (4.3%)	2,880 (4.6%)	4,200 (4.5%)	49.5%	2,139 (4.5%)	2,061 (4.6%)
Asian	1.2%	2,821 (1.4%)	811 (1.3%)	1,179 (1.3%)	41.8%	613 (1.3%)	566 (1.3%)
Indigenous	0.7%	1,309 (0.7%)	194 (0.3%)	608 (0.7%)	46.4%	460 (1.0%)	148 (0.3%)
**Central-South (*****n*** **=** **640,916)**							
White	58.7%	406,146 (63.4%)	136,622 (64.1%)	149,169 (62.6%)	36.7%	66,164 (61.2%)	83,005 (63.7%)
Mixed-race	33.2%	188,455 (29.4%)	61,157 (28.7%)	69,921 (29.3%)	37.1%	32,456 (30.0%)	37,465 (28.7%)
Black	6.8%	37,682 (5.9%)	12,655 (5.9%)	16,244 (6.8%)	43.1%	8,076 (7.5%)	8,168 (6.3%)
Asian	1.1%	7 591 (1.2%)	2,564 (1.2%)	2,721 (1.1%)	35.8%	1,175 (1.1%)	1,546 (1.2%)
Indigenous	0.3%	1,042 (0.2%)	295 (0.1%)	361 (0.2%)	34.6%	171 (0.2%)	190 (0.1%)

*Census of the Brazilian population.

Regarding patients that have comorbidities, there were more registered deaths in the North macro-region in all comorbidities compared to the Central-South. Anova test (*p* < 0.0001 for all comorbidities; F value for each comorbidity in the Supplemental Material), suggesting structural health disparities. Even with a different number of hospitalized patients and death in the regions, the previous quantities are relative to their population, which makes the comparison statistically plausible.

The older patients had a greater mortality percentage, especially in the North (*p* < 0.0001; *F* = 17489.1). In that region the non-survivors people over 51 years old are 55.8% and in Central-South they are 44.9%.

Table 2 shows a higher proportion of hospitalized deaths in the North (46.7%) than Central-South (37.2%), suggesting a regional effect. Anova test (*p* < 0.0001; *F* = 5755.17), shown in the Supplemental Material

In addition, there were higher proportions of black ethnicities in deaths in the Central-South region (43.1%), suggesting an ethnic effect. Anova test (*p* < 0.0001; *F* = 153.00) shown in the Supplemental Material with the Tukey test result.

**Figure 3:**
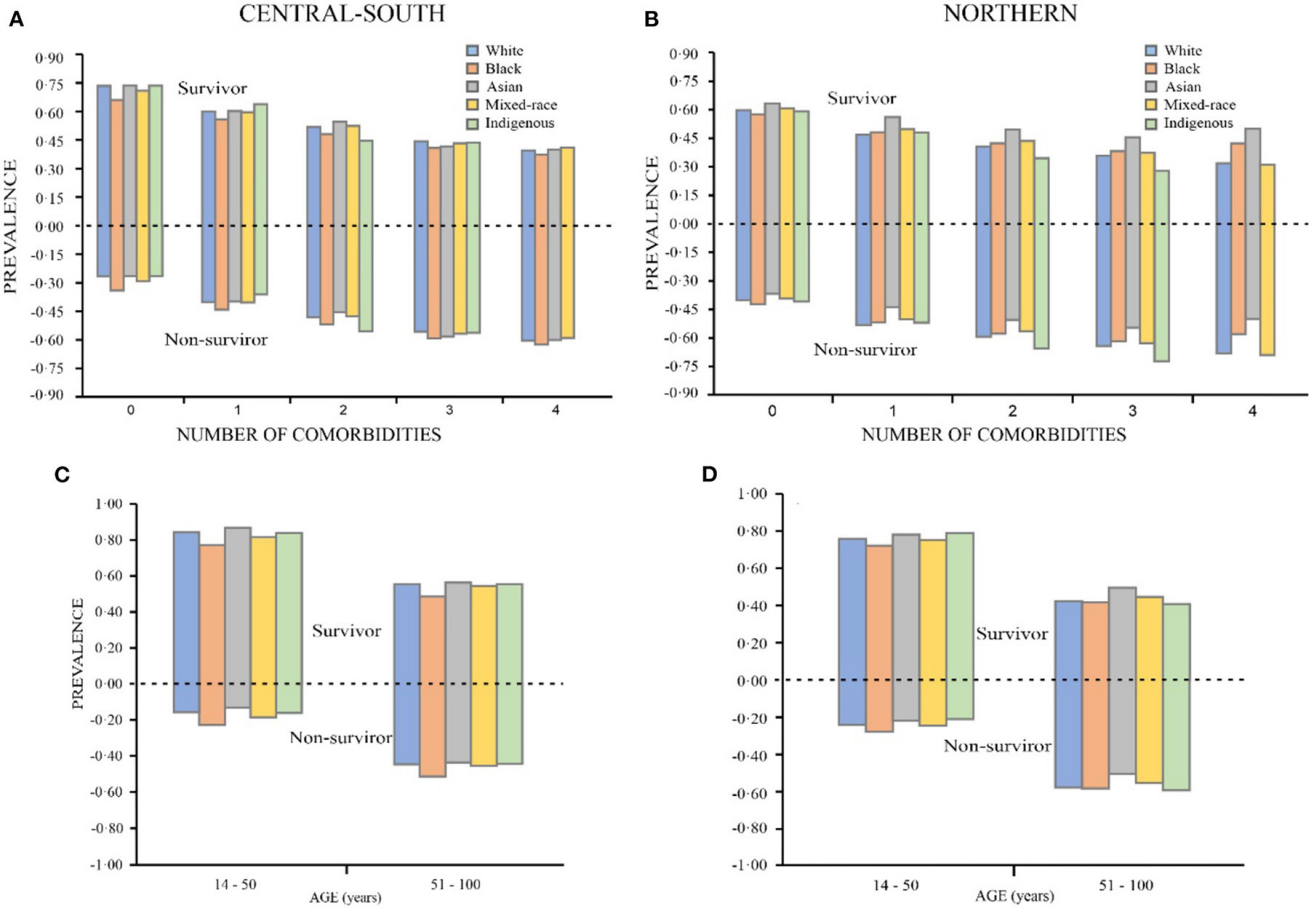
Ethnicity distributions according to number of comorbidities **(A,B)**, age group **(C,D)**.

Figures 3A,B show comorbidity prevalence calculated considering patient, ethnicity, and region, in this case, the Northern or Central-South, for both COVID survivors and deaths. One can see that there is substantial asymmetry between the results, with more deaths in the North. Furthermore, black Brazilians without comorbidities had the highest death rates in both the North (0.34) and the Central-South (0.42).

For groups with comorbidities in the North, most deaths were among indigenous (0.67) ethnicities. In the Central-South, when patients had one or more comorbidity, most deaths were among the black people (0.54).

The distribution of non-survivors, by age and ethnicity, that was higher for patients aged above 51 in the North (0.56) and Central-South (0.46), as shown Figures 3C,D. For patients aged 14–50, there was greater probability for survival in both regions. Analyzing only the descriptive data, one cannot determine large disparities in survival rates among racial groups according to number of comorbidities and age group.

The statistical model allowed us to observe which variables, and to what degree, they influence the individual's odds of mortality based on the coefficient obtained. The validity of the model is shown in Table 3, with *p* < 0.0001 for all variables, showing that they are explanatory for calculating the probability of an individual not surviving the disease.

**Table 3:** Model for mortality rates.

**Parameter**		**Estimate**	**Wald qui-square**	**Pr > ChiSq**
Intercept		−1.5534	4143.2854	<0.0001
Age_less_50_years		−0.8672	2678.2748	<0.0001
Ethnicity	Non-white	0.0603	128.3516	<0.0001
Gender	Male	0.1614	1127.7744	<0.0001
Cardiovascular_disease		0.1729	1114.5035	<0.0001
Diabetes		0.1875	1137.8771	<0.0001
Hematological_disease		0.2217	59.5684	<0.0001
Down_syndrome		0.2888	37.9069	<0.0001
Region	Northern	0.3127	2061.7896	<0.0001
Age_50_years_higher		0.4106	652.1627	<0.0001
Obesity		0.4556	2596.4568	<0.0001
Illiterate_patient		0.4566	1096.2094	<0.0001
Pulmonary_disease		0.4858	1513.6012	<0.0001
Liver_disease		0.5021	376.4814	<0.0001
Immunosuppresions		0.5278	1157.7189	<0.0001
Renal_disease		0.6774	2906.2803	<0.0001
Neurological_disease		0.6880	3007.4068	<0.0001
I_relative_income*		0.8959	1029.9471	<0.0001
Number of observations used			840,201	

*I relative income means the inverse of the relative income. The higher that indicator, the worse the individual's economic condition.

One can infer that the probability of a patient not surviving are greater for patients with some comorbidities, arranged in order of least to greatest, in Table 3. The three worst factors are neurological diseases, renal diseases, and immunodeficiency disorders. Among the social variables analyzed separately, illiterate patients are more likely to die from the disease, along with patients aged over 50. Patients residing in the Northern macro-region have coefficients that indicate a probability of mortality relative to patients in the Central-South regions, along with women relative to men. The relative income variable, relevant and central to this work, indicates that the higher the index, the lower the chance of patient death.

**Figure 4:**
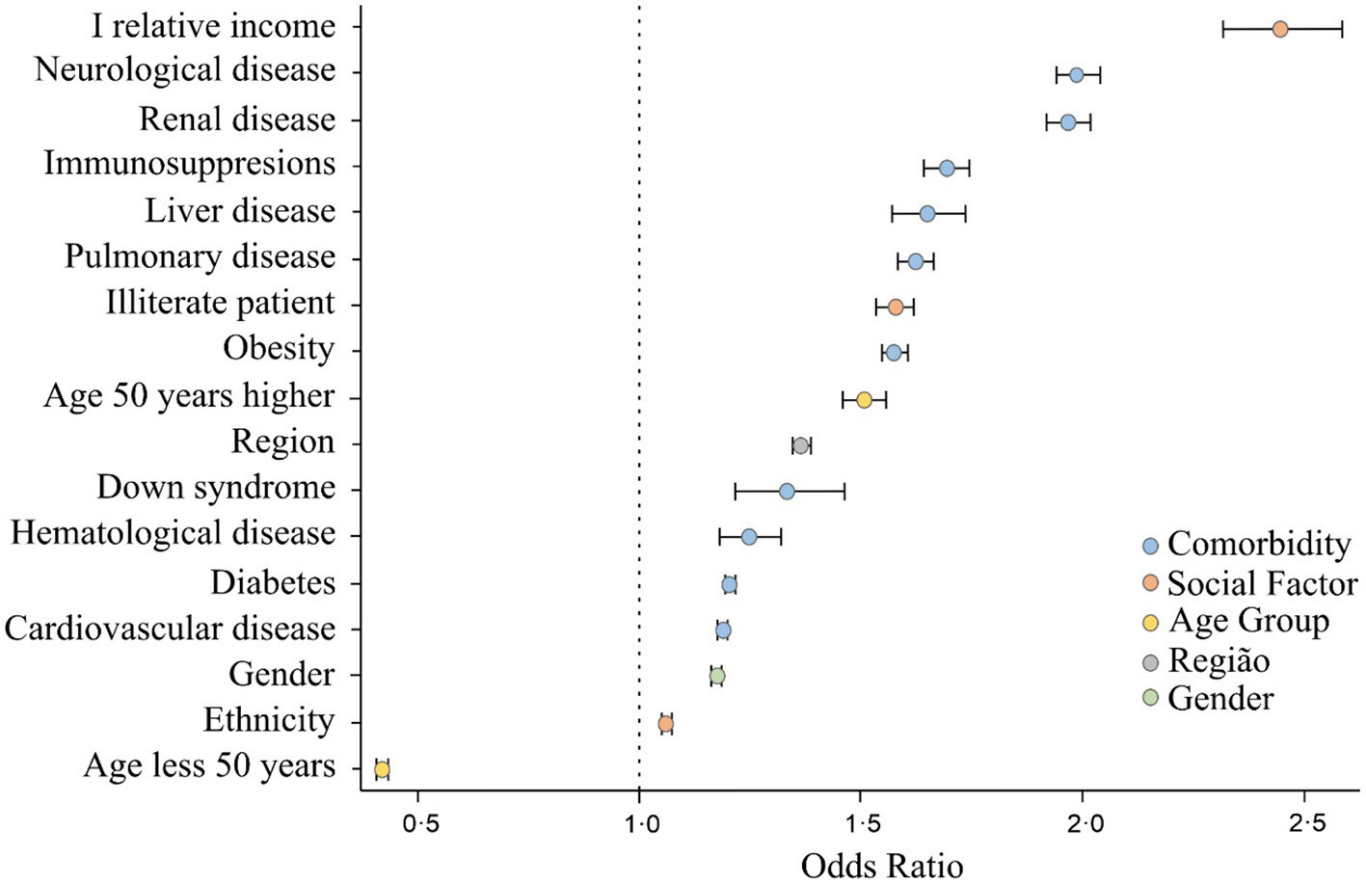
Social and risk factor indicators that lead to mortality for patients hospitalized with COVID-19.

Figure 4 shows the odds ratio for each variable, statistically significant at a 99% confidence interval. According to these indexes, the three comorbidities that most lead the patients to death are neurological disease, renal disease, and immunodeficiency disorders, increased by 1.99, 1.97, and 1.69 times, respectively.

One can see that social factors are very significant in increasing risk of death among hospitalized patients. For individuals with lower relative income, for example, odds of death increased 2.45 times. Also, regional factors are relevant, since patients residing in the North and Northeast regions of Brazil are 1.37 times more likely to die, possibly associated with the urgent care and emergency hospital conditions available to patients.

## Discussion

This paper presented a study on patient mortality for hospitalizations with COVID-19 in Brazil. To the best of our knowledge, this study gives the highest number of deaths from COVID-19 from both health and social factors using Brazilian data. We were able to verify that social factors were very decisive in determining COVID-19 mortality in Brazil, even higher than the comorbidity factors.

This analysis allowed us to observe that patient survival is higher for younger females with fewer comorbidities, in line with results worldwide (22–24). Also, other important and new trends for social factors were found in Brazil. Similar to Baqui et al. (11), significant regional variations were found both in terms of case characteristics and results. São Paulo, Minas Gerais, and Rio de Janeiro states had the most absolute cases. Amazonas, Rio Grande do Sul, and Santa Catarina sates had the most cases per 100,000 inhabitants. These states are important gateways to Brazil.

The distribution of hospitalized patients with COVID-19, i.e., the North, at 24% of all patients, and the Central-South, at 76%, is disproportional with the population sizes of these regions. The North holds 36% of the Brazilian population, and the Central-South region 64% (14). This difference highlights national heterogeneity, and we can therefore hypothesize that hospitalization rates in the North are lower than the Central-South, although this considers disproportionate increases of patients with COVID-19 in Amazonas in January 2021, in a new wave of cases following a new variant of the virus (25), leading us to infer that the hospital structure in the North was inadequate in attending patients.

Some substantial index variations were observed in each region. States in the Northern macro-region tended to have higher risk factors than states in the Central-South region, in alignment with the highest percentage of deaths in the North, according to Table 1. Many black Brazilians identify themselves as mixed-race (26). According to the analysis, both ethnic groups share high-risk factors and high death rates.

Of all patients diagnosed with COVID-19 (hospitalized or not) who died in Brazil, the Central-South region has the highest mortality rate per 100,000 inhabitants (27). However, when the mortality rate is calculated for hospitalized patients only, the numbers are different. The logistic regression results suggested that risk of death is 1.37 times greater in the Northern macro-region than in the Central-South macro-region when a patient is admitted to hospital with COVID-19. It strongly indicates the high complexity health system in the Central-South macro-region. That macro-region is the richer part of the country, despite also have great incoming and poverty inequality.

Additionally, the study corroborates information stating that the Brazilian risk group comprises the elderly with comorbidities (28). Furthermore, it has been demonstrated that hospitalized Brazilian black or mixed-race patients, or those who live in the North, are at greater risk of death from COVID-19. In short, relative income associated with race, illiteracy, and regional issues, are relevant indicators for social aspects related to the COVID-19 pandemic.

In Brazil, measures to contain the spread of the virus were managed by local cities. However, concerns about mortality rates are still growing, especially after confirmation of the first case of a new variant from India in Maranhão state on May 22, 2021, which is 50 times more communicable (29). Across the country, urgent political attention is required, directed toward the importance of vaccination in preventing COVID-19 mortality, especially when the official number of deaths has surpassed the 600,000 mark in Brazil.

The estimated relative income index was the most intense result in the statistical model performed, demonstrating that the worse the socioeconomic conditions of the individuals, the greater their chance of death when hospitalized with COVID-19. This index (relative income) was even greater than the main comorbidity found (neurological disease).

Additionally, individuals living in less populated regions located further North of the country were also more likely to die when they required hospitalization. This information led us to conclude that it is essential to strengthen the conditions of high-complexity care in the public health system, especially for the poorest individuals. Besides, it is important to consider in the public health system planning, more equitable distribution of equipment and medical support.

This study had some limitations especially regarding to the existing database. To avoid missing information, we clipped 43.5% from the initial database. These records were about evolution, ethnicity and, gender. Besides, the social database does not consider people under 14 years old, which exclude the possibility to analyze this group. We suggest that in future studies this could be better explored and considered.

## Data Availability Statement

Publicly available datasets were analyzed in this study. This data can be found at: https://opendatasus.saude.gov.br/dataset/bd-srag-2021.

## Ethics Statement

Ethical review and approval was not required for the study on human participants in accordance with the local legislation and institutional requirements. Written informed consent from the participants' legal guardian/next of kin was not required to participate in this study in accordance with the national legislation and the institutional requirements.

## Author Contributions

WR, DP, DT, and RR developed the research question and reviewed the statistical results. DT and HC collected and pre-processed the epidemiological and socioeconomic data. WR, DP, DT, and HC conducted analysis with descriptive statistics and regression logistic. GR participated in the theoretical foundations. All authors designed the study, analysis plan, reviewed early, and final versions of this manuscript.

## Conflict of Interest

The authors declare that the research was conducted in the absence of any commercial or financial relationships that could be construed as a potential conflict of interest.

## Publisher's Note

All claims expressed in this article are solely those of the authors and do not necessarily represent those of their affiliated organizations, or those of the publisher, the editors and the reviewers. Any product that may be evaluated in this article, or claim that may be made by its manufacturer, is not guaranteed or endorsed by the publisher.
