# Supplementary material for: Social, Economic, and Regional Determinants of Mortality in Hospitalized Patients With COVID-19 in Brazil
Source: Front Public Health. 2022 Mar 31;10:856137. doi: 10.3389/fpubh.2022.856137 (PMC9008306; doi:10.3389/fpubh.2022.856137)
Supplement: Supplementary file 1 [file Data_Sheet_1.pdf]

*Supplementary Material*

# 1 Supplementary Data

## 1.1 Hypothesis tests results

**Macro-regions**

The test shows that there were more deaths in the Northern macro-region.
*Consider N for Northern and S for Central-South macro-region.

| Source | DF | Sum of Squares | Mean Square | F Value | Pr > F |
|---|---|---|---|---|---|
| Model | 1 | 1365.3510 | 1365.3510 | 5755.17 | <.0001 |
| Error | 840199 | 199328.1249 | 0.2372 | | |
| Corrected Total | 840200 | 200693.4760 | | | |

| R-Square | Coeff Var | Root MSE | deaths Mean |
|---|---|---|---|
| 0.006803 | 123.4747 | 0.487072 | 0.394471 |

| Source | DF | Anova SS | Mean Square | F Value | Pr > F |
|---|---|---|---|---|---|
| macro_region | 1 | 1365.351033 | 1365.351033 | 5755.17 | <.0001 |

The ANOVA Procedure

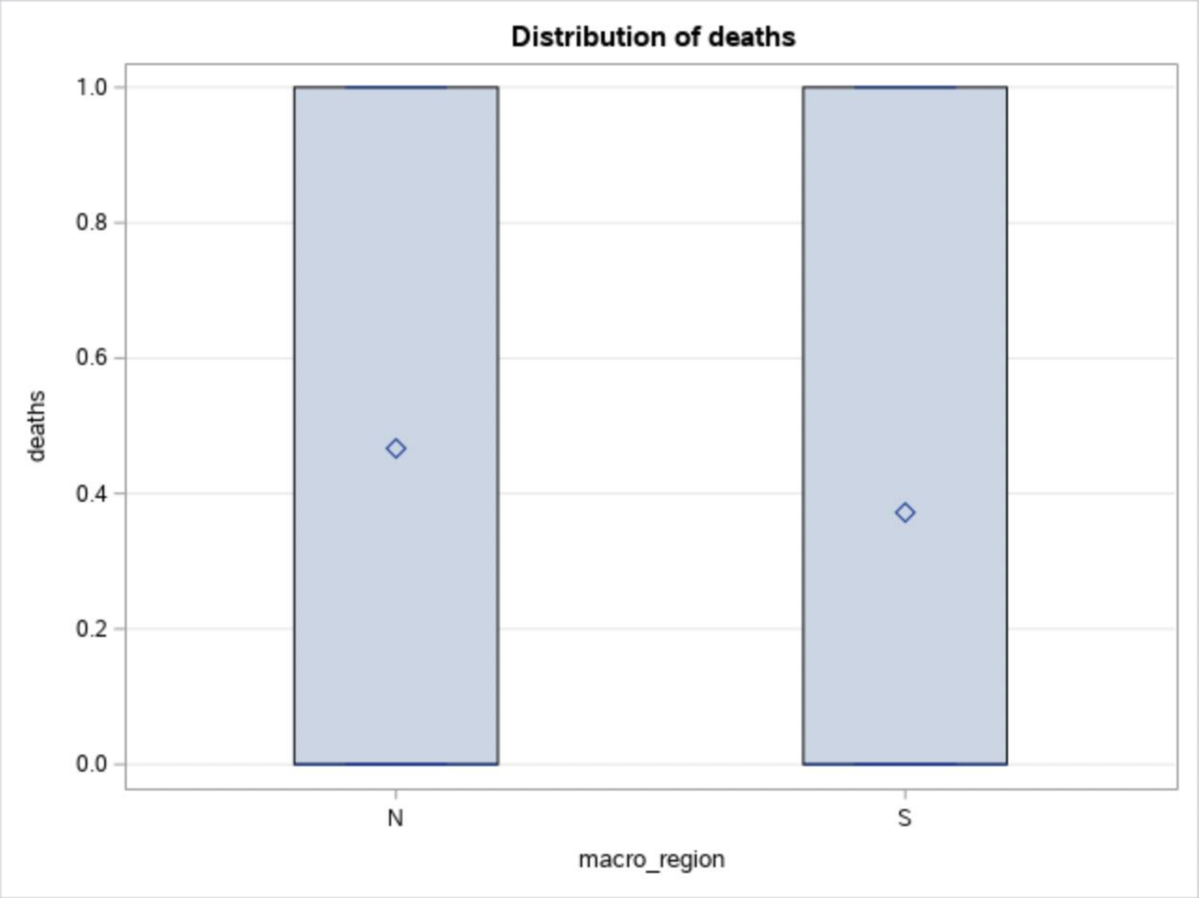


**Supplementary Figure 1 Difference between Macro-regions Central-South and North**

**<u>Ethnicity</u>**
The test shows that were higher proportions of black ethnicities in deaths in the Central-South region.
*Consider the following values to each ethnicity:
1: White
2: Black
3: Asian
4: Mixed-race
5: Indigenous

| Source | DF | Sum of Squares | Mean Square | F Value | Pr > F |
|---|---|---|---|---|---|
| Model | 4 | 142.8372 | 35.7093 | 153.00 | <.0001 |
| Error | 640911 | 149584.1785 | 0.2334 | | |
| Corrected Total | 640915 | 149727.0157 | | | |

| R-Square | Coeff Var | Root MSE | deaths Mean |
|---|---|---|---|
| 0.000954 | 129.8703 | 0.483108 | 0.371993 |

| Source | DF | Anova SS | Mean Square | F Value | Pr > F |
|---|---|---|---|---|---|
| Ethnicity_South | 4 | 142.8372286 | 35.7093071 | 153.00 | <.0001 |

The ANOVA Procedure

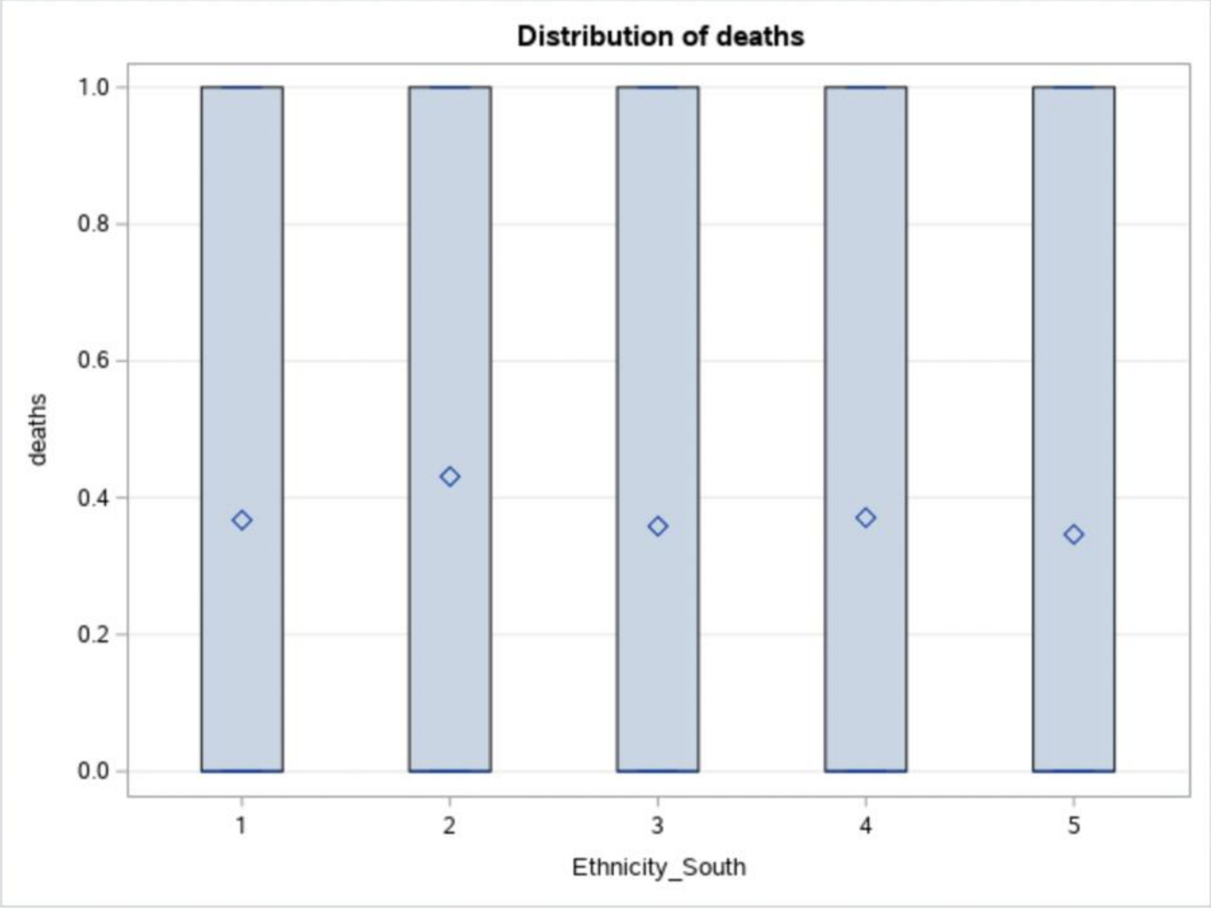


**Supplementary Figure 2 Deaths by ethnicity**

**Tukey test results:**

| Comparisons significant at the 0.05 level are indicated by ***. | | | | |
|---|---|---|---|---|
| **Ethnicity_South Comparison** | **Difference Between Means** | **Simultaneous 95% Confidence Limits** | | |
| Black – Mixed-race | 0.060059 | 0.052622 | 0.067495 | *** |
| Black – White | 0.063802 | 0.056705 | 0.070899 | *** |
| Black – Asian | 0.072630 | 0.056051 | 0.089209 | *** |
| Black – Indigenous | 0.084632 | 0.043247 | 0.126017 | *** |
| Mixed-race – White | 0.003743 | 0.000070 | 0.007416 | *** |
| Mixed-race – Asian | 0.012571 | -0.002855 | 0.027998 | |
| Mixed-race – Indigenous | 0.024573 | -0.016364 | 0.065510 | |
| White – Asian | 0.008828 | -0.006438 | 0.024094 | |
| White – Indigenous | 0.020830 | -0.020047 | 0.061707 | |
| Asian – Mixed-race | -0.012571 | -0.027998 | 0.002855 | |
| Asian – White | -0.008828 | -0.024094 | 0.006438 | |
| Asian – Indigenous | 0.012002 | -0.031535 | 0.055538 | |

**Supplementary Table 1 Tukey test results**

## Age

The test shows that the older patients had a greater mortality, especially in the North.
*Consider 0 representing patients under 51-year-old and 1 representing patients equal or over 51-year-old.

| Source | DF | Sum of Squares | Mean Square | F Value | Pr > F |
|---|---|---|---|---|---|
| Model | 1 | 4001.79452 | 4001.79452 | 17489.1 | <.0001 |
| Error | 199283 | 45599.31471 | 0.22882 | | |
| Corrected Total | 199284 | 49601.10924 | | | |

| R-Square | Coeff Var | Root MSE | deaths Mean |
|---|---|---|---|
| 0.080680 | 102.4819 | 0.478348 | 0.466764 |

| Source | DF | Anova SS | Mean Square | F Value | Pr > F |
|---|---|---|---|---|---|
| Age_North | 1 | 4001.794523 | 4001.794523 | 17489.1 | <.0001 |

The ANOVA Procedure

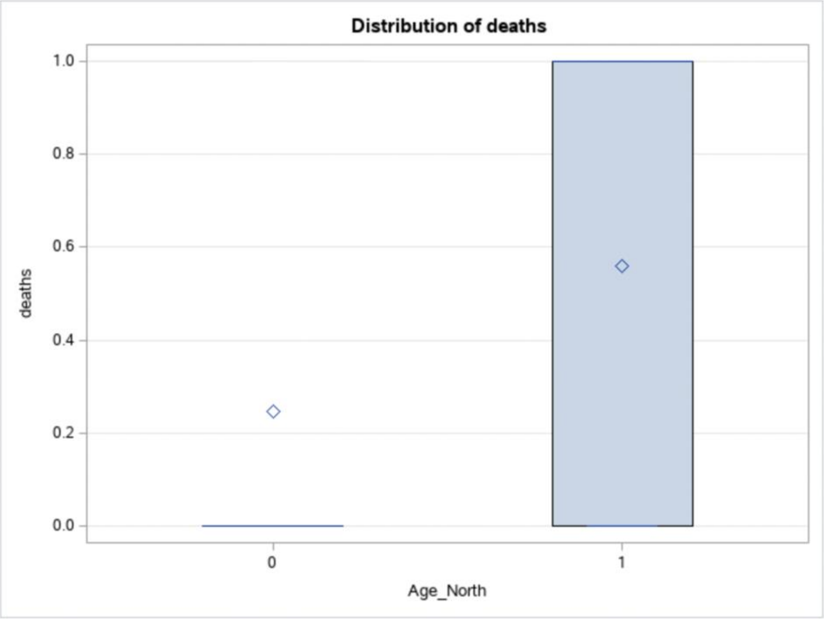


**Supplementary Figure 3 Mortality in Northern macro-region by age**

| Source | DF | Sum of Squares | Mean Square | F Value | Pr > F |
|---|---|---|---|---|---|
| Model | 1 | 10442.3395 | 10442.3395 | 48050.1 | <.0001 |
| Error | 640914 | 139284.6762 | 0.2173 | | |
| Corrected Total | 640915 | 149727.0157 | | | |

| R-Square | Coeff Var | Root MSE | deaths Mean |
|---|---|---|---|
| 0.069743 | 125.3192 | 0.466178 | 0.371993 |

| Source | DF | Anova SS | Mean Square | F Value | Pr > F |
|---|---|---|---|---|---|
| Age_South | 1 | 10442.33955 | 10442.33955 | 48050.1 | <.0001 |

The ANOVA Procedure

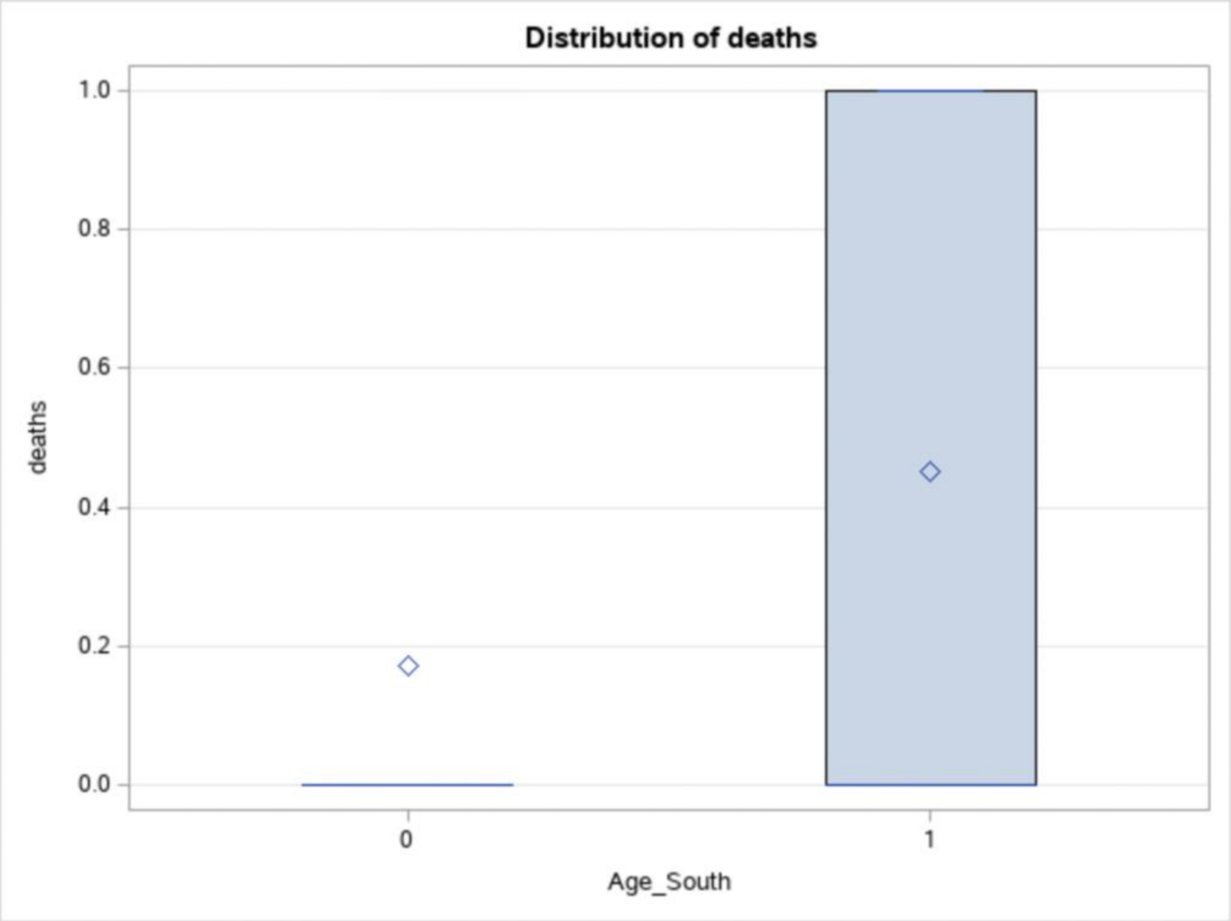


**Supplementary Figure 4 Mortality in Central-South macro-region by age**

**Comorbidities**

The test shows that there were more deaths in the North in all comorbidities.
*Consider 0 representing Central-South and 1 representing North macro-region.

| Source | DF | Sum of Squares | Mean Square | F Value | Pr > F |
|---|---|---|---|---|---|
| Model | 1 | 555.0144 | 555.0144 | 2330.00 | <.0001 |
| Error | 840199 | 200138.4615 | 0.2382 | | |
| Corrected Total | 840200 | 200693.4760 | | | |

| R-Square | Coeff Var | Root MSE | death Mean |
|---|---|---|---|
| 0.002765 | 123.7254 | 0.488061 | 0.394471 |

| Source | DF | Anova SS | Mean Square | F Value | Pr > F |
|---|---|---|---|---|---|
| renal | 1 | 555.0144454 | 555.0144454 | 2330.00 | <.0001 |

The ANOVA Procedure

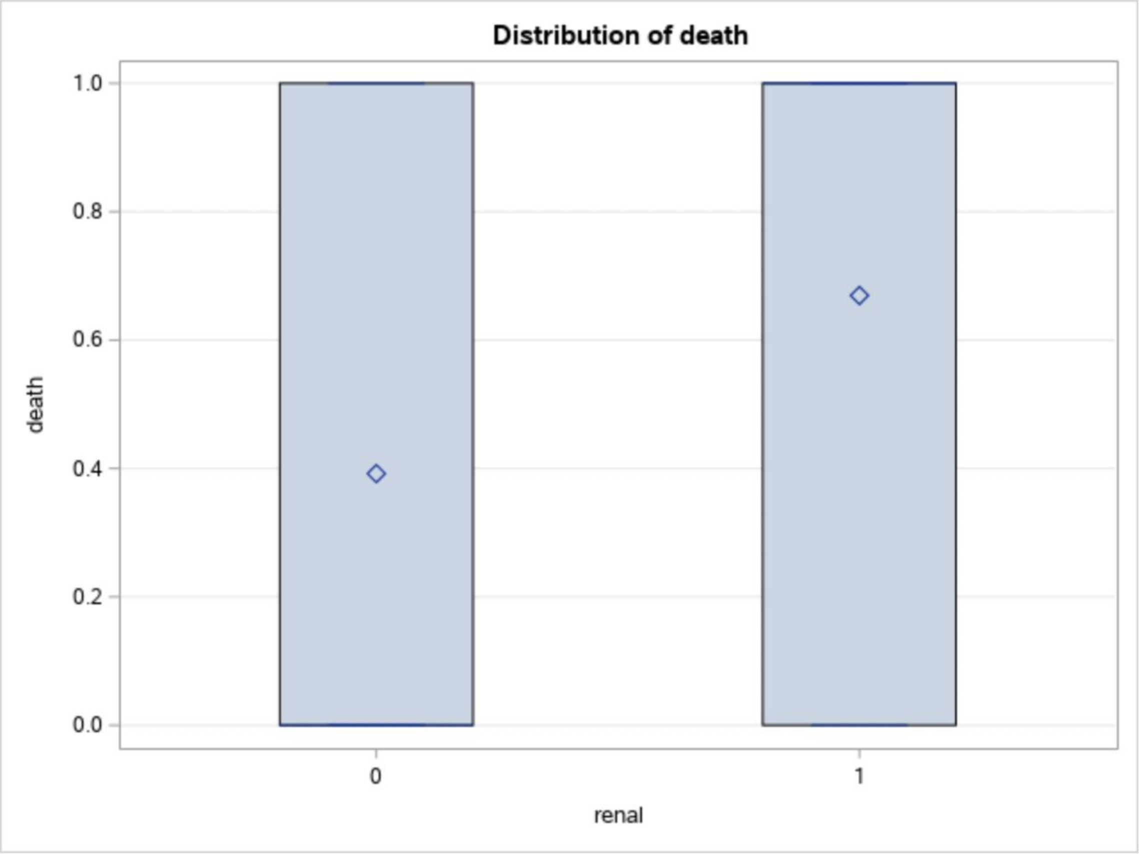


**Supplementary Figure 5 Renal disease anova test**

| Source | DF | Sum of Squares | Mean Square | F Value | Pr > F |
|---|---|---|---|---|---|
| Model | 1 | 631.5789 | 631.5789 | 2652.44 | <.0001 |
| Error | 840199 | 200061.8971 | 0.2381 | | |
| Corrected Total | 840200 | 200693.4760 | | | |

| R-Square | Coeff Var | Root MSE | death Mean |
|---|---|---|---|
| 0.003147 | 123.7018 | 0.487968 | 0.394471 |

| Source | DF | Anova SS | Mean Square | F Value | Pr > F |
|---|---|---|---|---|---|
| Liver | 1 | 631.5788973 | 631.5788973 | 2652.44 | <.0001 |

The ANOVA Procedure

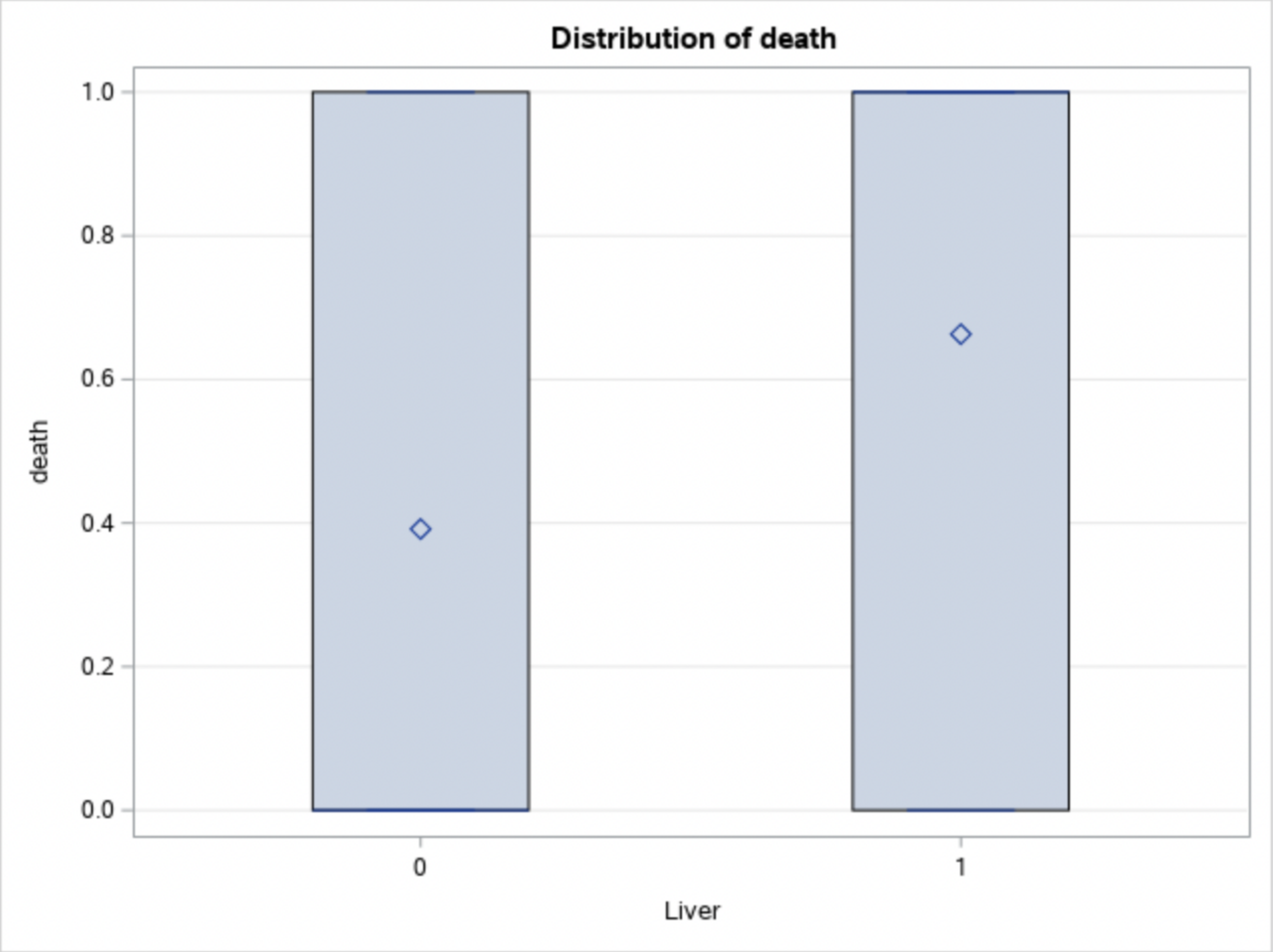


**Supplementary Figure 6 Liver disease anova test**

| Source | DF | Sum of Squares | Mean Square | F Value | Pr > F |
|---|---|---|---|---|---|
| Model | 1 | 129.5317 | 129.5317 | 542.63 | <.0001 |
| Error | 840199 | 200563.9443 | 0.2387 | | |
| Corrected Total | 840200 | 200693.4760 | | | |

| R-Square | Coeff Var | Root MSE | death Mean |
|---|---|---|---|
| 0.000645 | 123.8569 | 0.488580 | 0.394471 |

| Source | DF | Anova SS | Mean Square | F Value | Pr > F |
|---|---|---|---|---|---|
| Immunosuppression | 1 | 129.5317117 | 129.5317117 | 542.63 | <.0001 |

The ANOVA Procedure

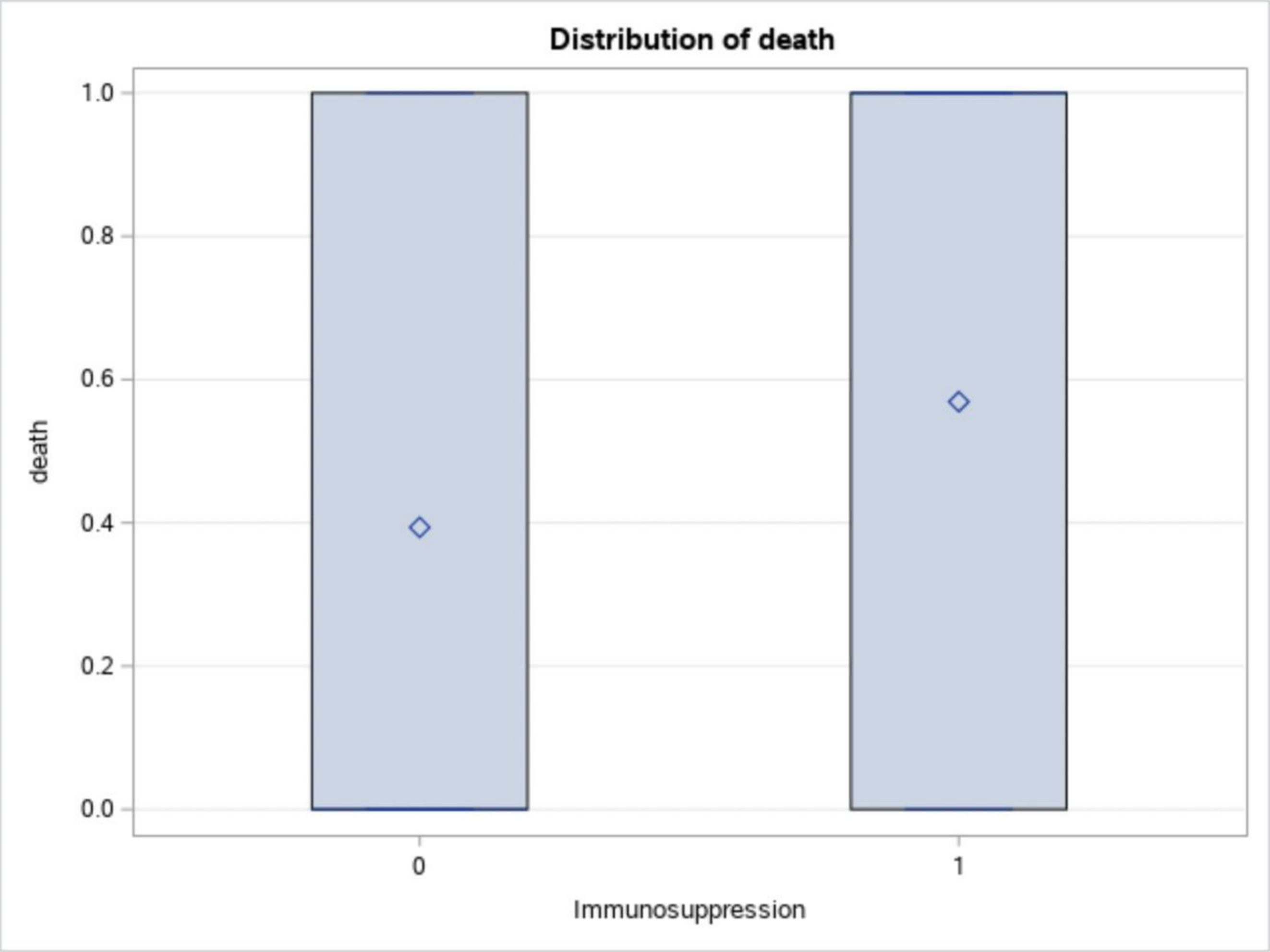


**Supplementary Figure 7 Immunosuppression disease anova test**

| Source | DF | Sum of Squares | Mean Square | F Value | Pr > F |
|---|---|---|---|---|---|
| Model | 1 | 210.0553 | 210.0553 | 880.31 | <.0001 |
| Error | 840199 | 200483.4206 | 0.2386 | | |
| Corrected Total | 840200 | 200693.4760 | | | |

| R-Square | Coeff Var | Root MSE | death Mean |
|---|---|---|---|
| 0.001047 | 123.8320 | 0.488482 | 0.394471 |

| Source | DF | Anova SS | Mean Square | F Value | Pr > F |
|---|---|---|---|---|---|
| obesity | 1 | 210.0553463 | 210.0553463 | 880.31 | <.0001 |

The ANOVA Procedure

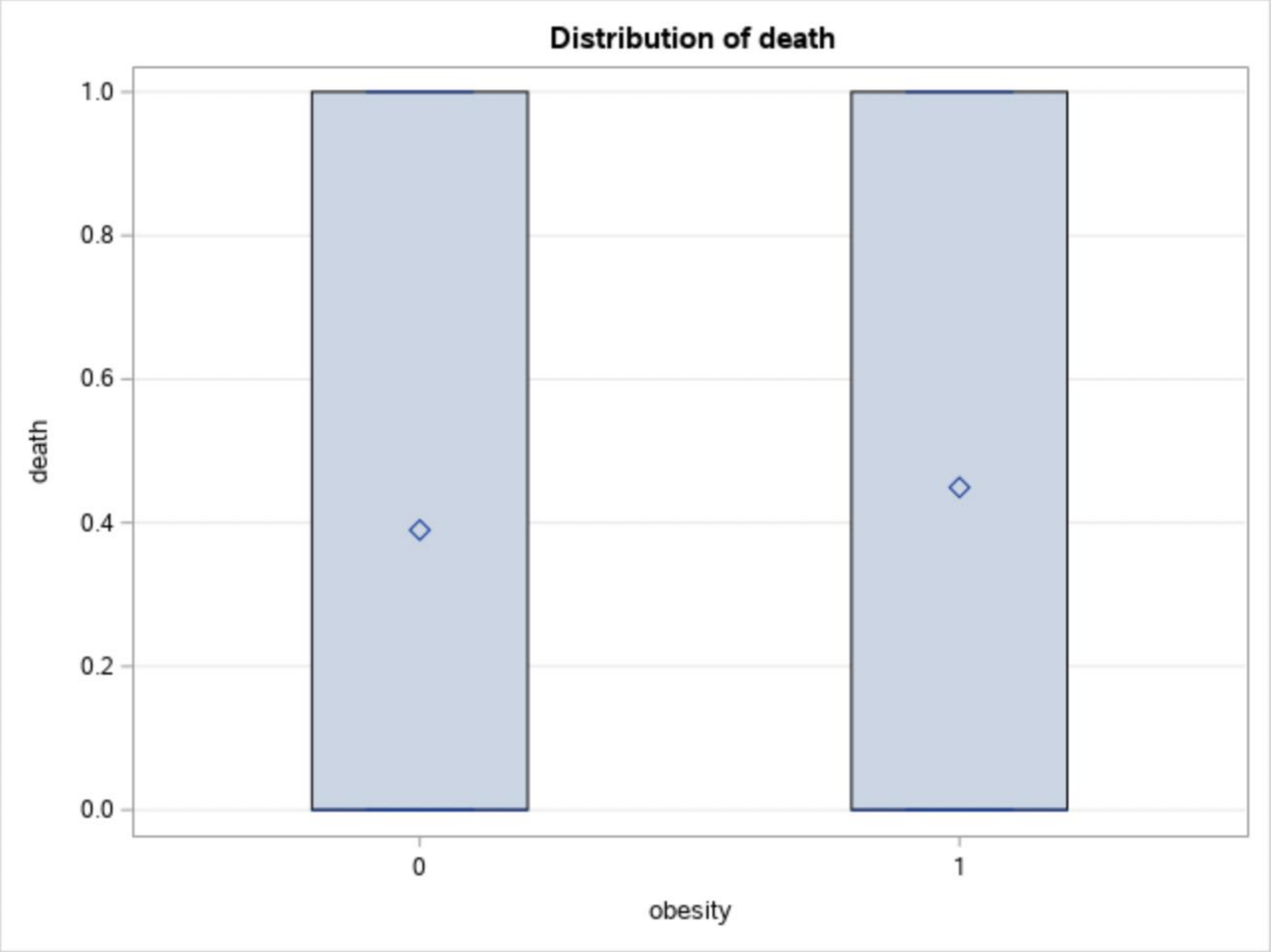


**Supplementary Figure 8 Obesity anova test**

| Source | DF | Sum of Squares | Mean Square | F Value | Pr > F |
|---|---|---|---|---|---|
| Model | 1 | 296.2204 | 296.2204 | 1241.95 | <.0001 |
| Error | 840199 | 200397.2556 | 0.2385 | | |
| Corrected Total | 840200 | 200693.4760 | | | |

| R-Square | Coeff Var | Root MSE | death Mean |
|---|---|---|---|
| 0.001476 | 123.8054 | 0.488377 | 0.394471 |

| Source | DF | Anova SS | Mean Square | F Value | Pr > F |
|---|---|---|---|---|---|
| Neurological | 1 | 296.2204038 | 296.2204038 | 1241.95 | <.0001 |

The ANOVA Procedure

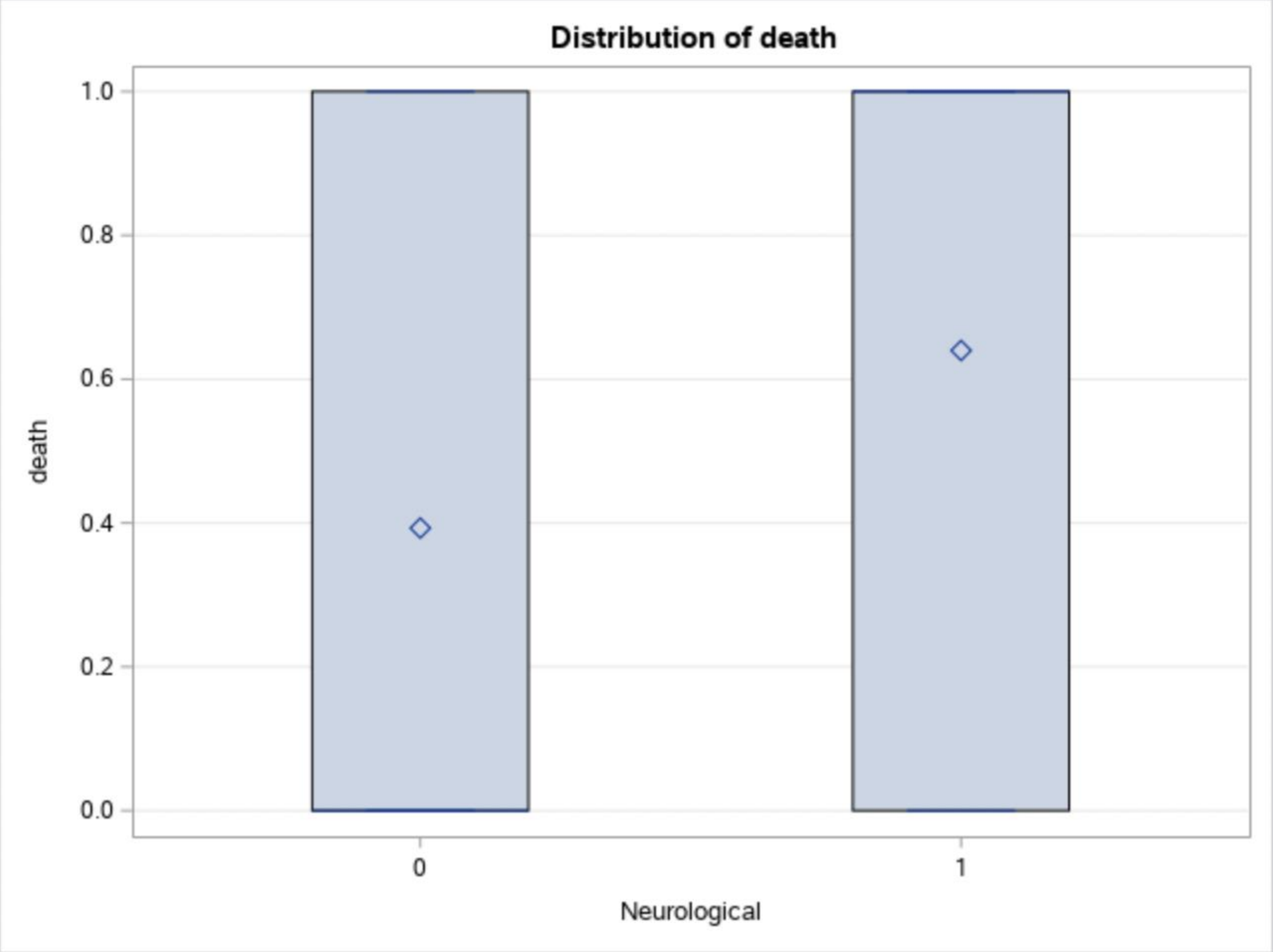


**Supplementary Figure 9 Neurological disease anova test**

| Source | DF | Sum of Squares | Mean Square | F Value | Pr > F |
|---|---|---|---|---|---|
| Model | 1 | 286.8598 | 286.8598 | 1202.65 | <.0001 |
| Error | 840199 | 200406.6161 | 0.2385 | | |
| Corrected Total | 840200 | 200693.4760 | | | |

| R-Square | Coeff Var | Root MSE | death Mean |
|---|---|---|---|
| 0.001429 | 123.8083 | 0.488388 | 0.394471 |

| Source | DF | Anova SS | Mean Square | F Value | Pr > F |
|---|---|---|---|---|---|
| Pulmonary | 1 | 286.8598372 | 286.8598372 | 1202.65 | <.0001 |

The ANOVA Procedure

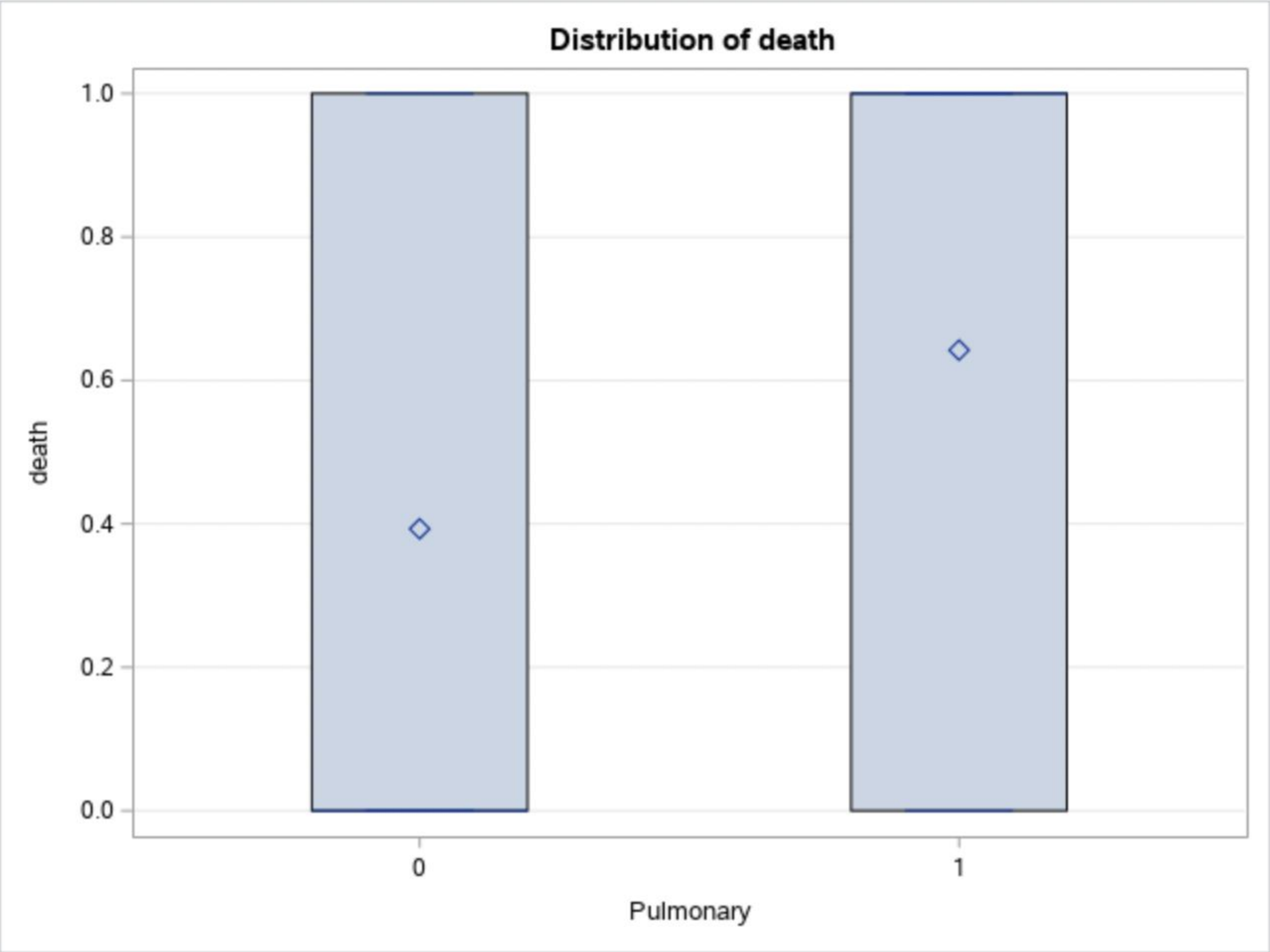


**Supplementary Figure 10 Pulmonary disease anova test**

| Source | DF | Sum of Squares | Mean Square | F Value | Pr > F |
|---|---|---|---|---|---|
| Model | 1 | 31.9240 | 31.9240 | 133.67 | <.0001 |
| Error | 840199 | 200661.5520 | 0.2388 | | |
| Corrected Total | 840200 | 200693.4760 | | | |

| R-Square | Coeff Var | Root MSE | death Mean |
|---|---|---|---|
| 0.000159 | 123.8870 | 0.488698 | 0.394471 |

| Source | DF | Anova SS | Mean Square | F Value | Pr > F |
|---|---|---|---|---|---|
| Hematologic | 1 | 31.92398222 | 31.92398222 | 133.67 | <.0001 |

The ANOVA Procedure

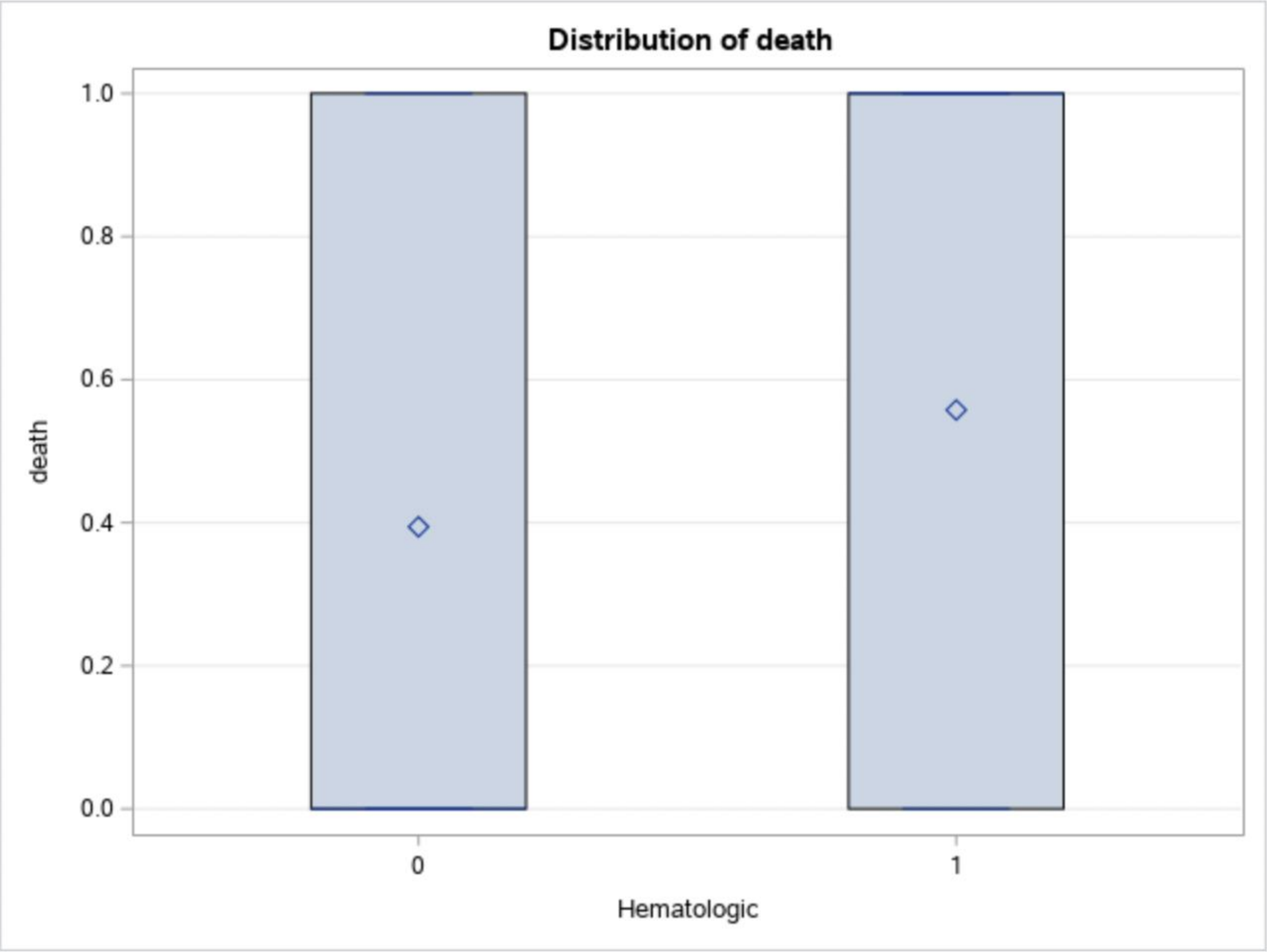


**Supplementary Figure 11 Hematologic disease anova test**

| Source | DF | Sum of Squares | Mean Square | F Value | Pr > F |
|---|---|---|---|---|---|
| Model | 1 | 1346.3941 | 1346.3941 | 5674.72 | <.0001 |
| Error | 840199 | 199347.0819 | 0.2373 | | |
| Corrected Total | 840200 | 200693.4760 | | | |

| R-Square | Coeff Var | Root MSE | death Mean |
|---|---|---|---|
| 0.006709 | 123.4806 | 0.487095 | 0.394471 |

| Source | DF | Anova SS | Mean Square | F Value | Pr > F |
|---|---|---|---|---|---|
| diabetes | 1 | 1346.394087 | 1346.394087 | 5674.72 | <.0001 |

The ANOVA Procedure

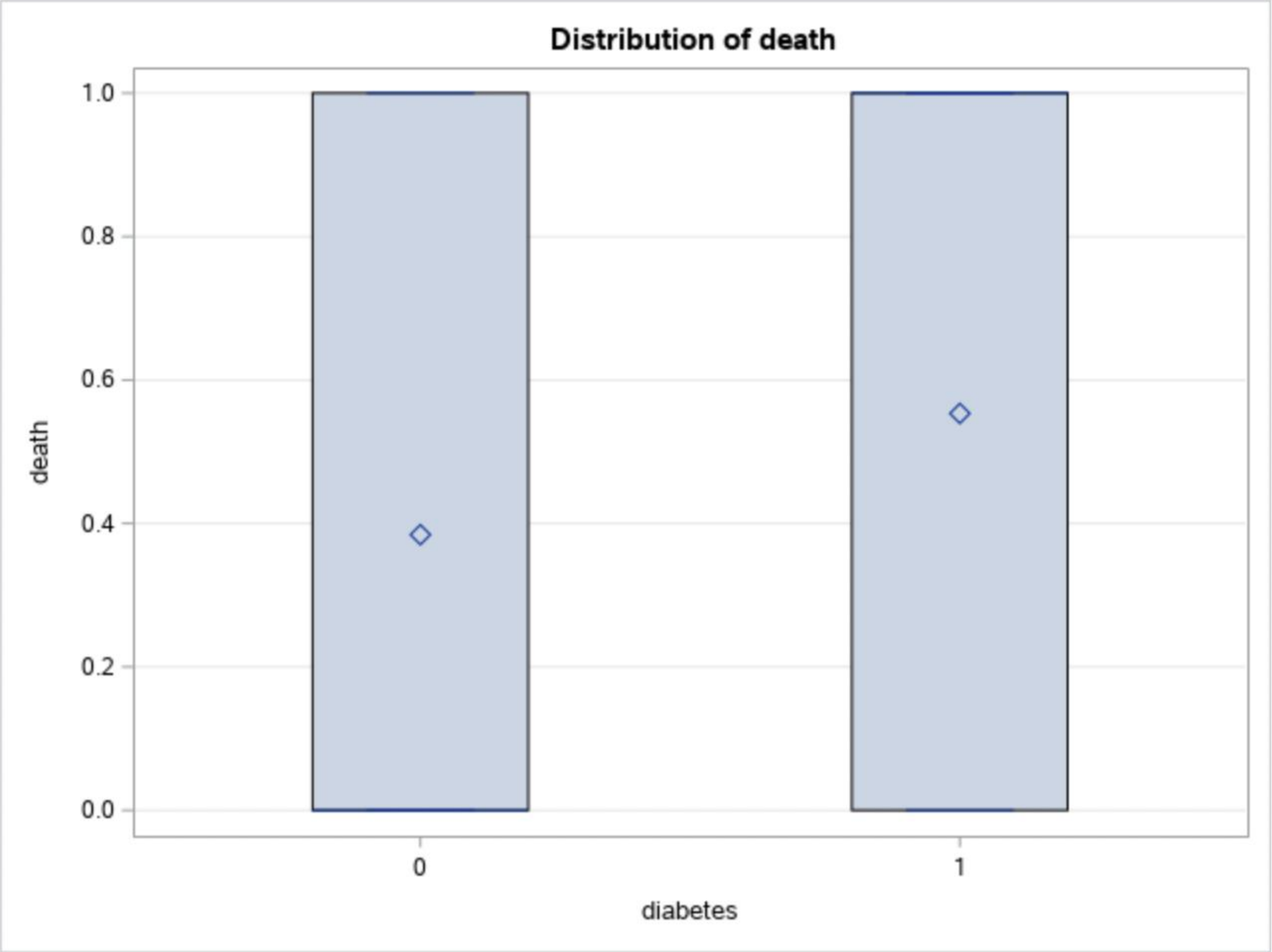


**Supplementary Figure 12 Diabetes anova test**

| Source | DF | Sum of Squares | Mean Square | F Value | Pr > F |
|---|---|---|---|---|---|
| Model | 1 | 1594.5487 | 1594.5487 | 6729.01 | <.0001 |
| Error | 840199 | 199098.9273 | 0.2370 | | |
| Corrected Total | 840200 | 200693.4760 | | | |

| R-Square | Coeff Var | Root MSE | death Mean |
|---|---|---|---|
| 0.007945 | 123.4037 | 0.486792 | 0.394471 |

| Source | DF | Anova SS | Mean Square | F Value | Pr > F |
|---|---|---|---|---|---|
| Cardiovascular | 1 | 1594.548717 | 1594.548717 | 6729.01 | <.0001 |

The ANOVA Procedure

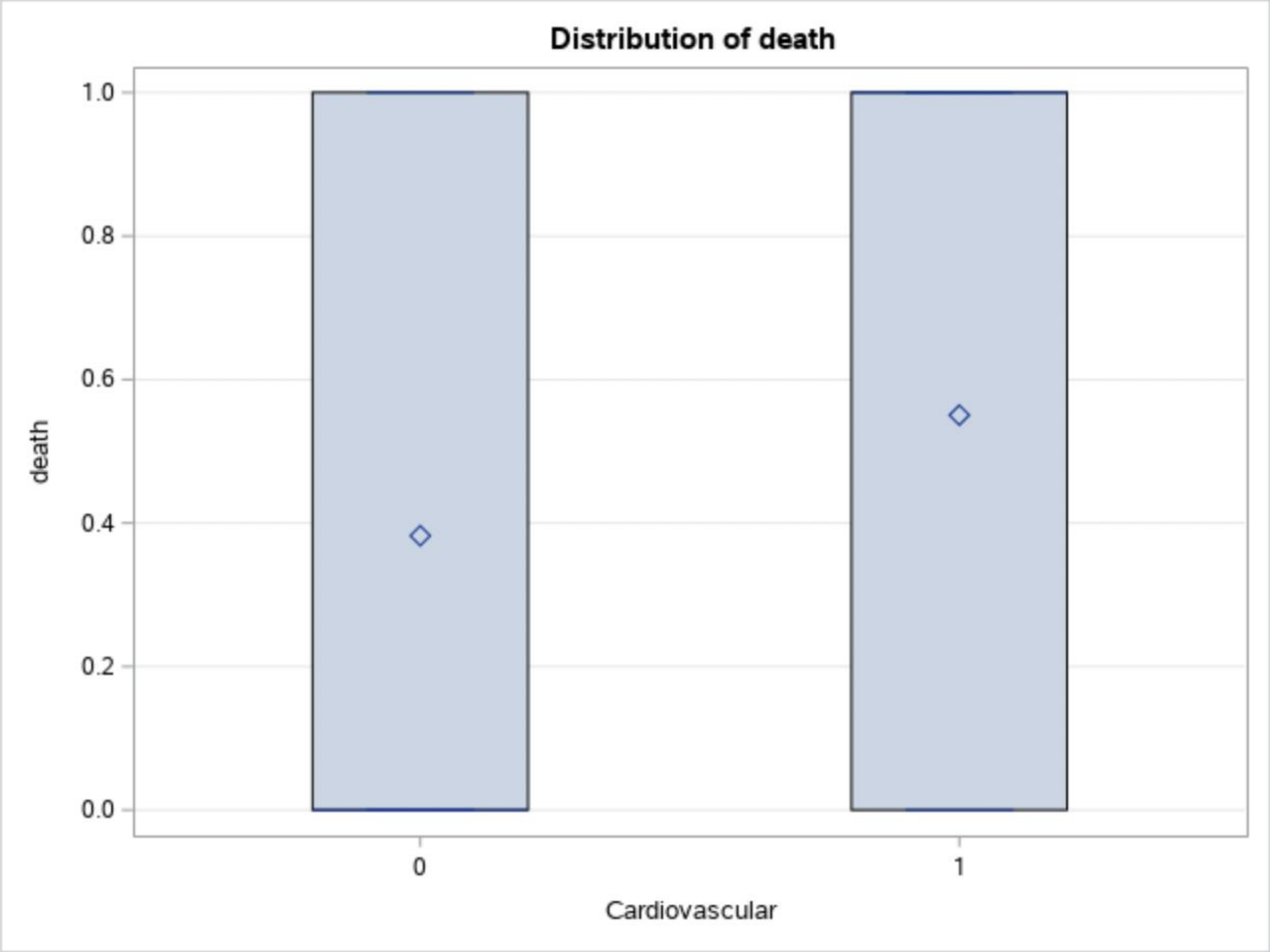


**Supplementary Figure 13 Cardiovascular disease anova test**

### 1.2 Code used to perform the Logistic Regression

```
LIBNAME mydata "/home/u49148566/ " access=readonly;
DATA new; set home.covid_paciente; /*database released on May/15/2021*/

if cs_raca in (2,3,4,5) then cs_raca = 0; /*Raça não branca*/

if macro_regiao = 'S' THEN macro_regiao = 1;
if macro_regiao = 'N' THEN macro_regiao = 0;
macro_regiao1 = input(macro_regiao, 4.); /*casting the variable*/

/*We selected the patients that were between 14 and 100 years old*/
if idade_paciente >= 14 or idade_paciente >100;

/*Divided the patients between Age_less_50_years and Age_50_years_higher*/
if idade_paciente <=50 then idade_menor_40 = 1;
if idade_paciente > 50 then idade_maior_50 = 1;

/*The relative income means the inverse of the relative income. The higher that indicator, the worse the individual's economic condition.*/
renda_relativa1 = 1-renda_relativa_p3;

/*Renaming the variables*/
RENAME evolucao = Death renal = Renal_disease macro_regiao1 = Region neurologic = Neurological_disease imunodepre = Immunosuppresions
       hepatica = Liver_disease pneumopati = Pulmonary_disease cs_escol_n_analfabeto = Illiterate_patient idade_maior_60 = Age_50_years_higher
       Obesidade = Obesity hematologi = Hematological_disease diabetes = Diabetes cs_sexo = Gender cardiopati = Cardiovascular_disease
       cs_raca = Ethnicity idade_menor_40 = Age_less_50_years renda_relativa1 = I_relative_income sind_down = Down_syndrome;

/*Applying the logistic regression*/
PROC LOGISTIC descending plots(only)=oddsratio(order = descending)simple ; /*range = (0,2)*/

class Ethnicity(ref='1') Gender(ref='F') Region(ref='1') / param=ref;

model Death = Age_50_years_higher Age_less_50_years Down_syndrome Region Neurological_disease Renal_disease Immunosuppresions
              Liver_disease Pulmonary_disease Illiterate_patient Obesity Hematological_disease Diabetes Gender Cardiovascular_disease
              Ethnicity I_relative_income / ctable;

run;
```
